# Progression and perspectives in disease modeling for Juvenile myelomonocytic leukemia

**DOI:** 10.1007/s12032-024-02549-5

**Published:** 2024-12-09

**Authors:** Shengyuan Fu, Yao Guo, Zhiyong Peng, Dengyang Zhang, Zhiguang Chang, Yan Xiao, Qi Zhang, Liuting Yu, Chun Chen, Yun Chen, Yuming Zhao

**Affiliations:** 1 Edmond H. Fischer Translational Medical Research Laboratory, Scientific Research Center, The Seventh Affiliated Hospital, Sun Yat-sen University, Shenzhen, 518107 Guangdong China; 2 Nanfang-Chunfu Children’s Institute of Hematology, Taixin Hospital, Dongguan, Guangdong China; 3 Department of Pediatrics, The Seventh Affiliated Hospital, Sun Yat-Sen University, Shenzhen, 518107 Guangdong China

**Keywords:** JMML, Preclinical research models, Primary cell culture, Cell models, Animal models

## Abstract

Juvenile myelomonocytic leukemia (JMML) is a rare myeloproliferative neoplasm occurring in infants and young children. JMML has been shown to be resistant to all conventional cytotoxic chemotherapy drugs, and current curative therapies still rely on hematopoietic stem cell transplantation, which carries a high risk of relapse post-transplantation. This underscores the urgent need for novel treatment strategies. However, the rarity of JMML poses a major limitation for research, as it is difficult to collect substantial primary research material. To gain a deeper insight into the underlying biological mechanisms of JMML, researchers are continuously improving and developing preclinical research models to better emulate the disease. Therefore, this review aims to delineate the various experimental models currently employed in JMML, including patient-derived cell-based models, cell models, and animal models. We will discuss the characterization of these models in the context of JMML, hoping to provide a valuable reference for researchers in this field.

## Introduction

Juvenile myelomonocytic leukemia (JMML) is classified and defined as a myeloproliferative neoplasm (MPN) [1]. Its annual incidence is approximately 1.2 per million children, and with a median age at diagnosis of only 2 years [2, 3].Clinical manifestations in children with JMML are related to infiltration of different organs by myelomonocytic cells, with most patients presenting with hepatosplenomegaly, fever [1]. Hematologic diagnostic criteria mainly include an increased peripheral blood (PB) monocyte count and a low percentage of PB and bone marrow (BM) blast cells [4]. It is noteworthy that in the diagnosis of JMML with special emphasis on the molecular detection, patients often need to carry one of the following mutations: somatic mutations in *PTPN11, NRAS, KRAS*, and germline mutations in *NF1* and *CBL* [1, 4]. As it is demonstrated that over 90% of JMML cases involve uncontrolled activation of the RAS pathway caused by these mutations. Aberrant RAS pathway activation is both a transforming event and a critical factor in disease progression. Specific gene mutations largely dictate the clinical manifestations and serve as important criteria for risk stratification. Research on JMML patients often revolves around different mutation spectra. Such analyses not only aid in comprehending the impact of various gene mutation types on the clinical manifestations and treatment responses of patients, but also provide crucial evidence for achieving more precise therapeutic interventions.

**Fig. 1:**
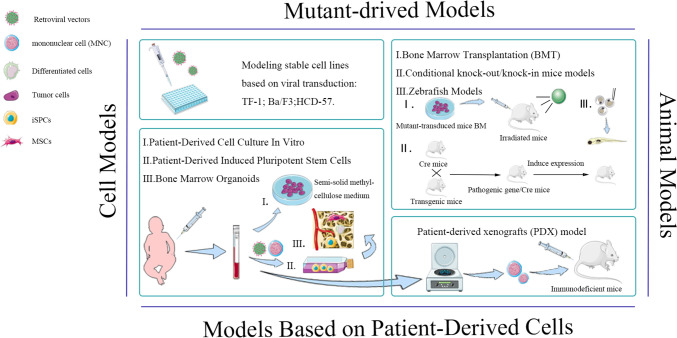
Schematics of different strategies for remodeling JMML. The disease models mainly include cell models, patient-derived cell-based models, and animal models

Currently, HSCT remains the primary treatment for JMML, but the 5-year survival rate of only 50%–60%. This underscores the imperative need for the development of novel treatment strategies. Given the widespread presence of DNA methylation abnormalities in JMML, the use of DNA methyltransferase inhibitors such as Azacitidine for epigenetic therapy has become an important part of treatment. Although promising, this approach still fails to be a curative treatment. One of the major hurdles in comprehensively studying JMML is its rarity, which makes it difficult to collect sufficient primary research material. Consequently, establishing preclinical research models that accurately mimic this disease is indispensable for facilitating more precise targeted therapeutic interventions. In the ensuing sections, we introduce several commonly employed models in JMML research, including patient-derived cell-based models, cell models, and animal models, and expose the characterization of each model in the context of JMML (Fig. 1).

## Patient-derived cells models

Unfortunately, immortalized cell lines derived from JMML patients have not been established to date [5]. Unlike acute leukemia, JMML cell does not exhibit mature arrest, making it almost impossible to generate stable immortalized cell lines [6]. Additionally, JMML primary cells undergo rapid differentiation and death in vitro, making long-term maintenance using traditional suspension culture methods challenging [7]. Particularly due to the extremely low incidence rate of JMML and the fact that the primary patient population is infants and young children, researchers face difficulties in obtaining a large number of cell samples for large-scale experimental studies. Over the past two decades, researches in genomics, transcriptomics, and epigenetics research based on JMML cell samples has significantly advanced our understanding of this disease. The analysis of JMML methylation has provided foundational support for the use of demethylating drugs [8]. Single-cell sequencing has revealed heterogeneous disease-propagating stem cells, uncovering the heterogeneity among different cell subtypes during JMML pathogenesis [9]. These advancements provide essential theoretical and practical foundations for the development of personalized treatment strategies. These also emphasize the importance of patient-derived cell in JMML research.

### Patient-derived cell culture in vitro

The initial investigation of JMML disease commenced with the cultivation of JMML primary cells in semi-solid methylcellulose culture medium [10]. Through this method, researchers made significant discoveries regarding the hypersensitivity of JMML progenitor cells to granulocyte–macrophage colony-stimulating factor (GM-CSF) and elucidated the pivotal role of Ras signaling transduction in JMML [11–13]. This approach has become a hallmark and important diagnostic tool for the disease. Furthermore, researchers have utilized this cell culture method to screen various potential drugs by assessing their inhibitory effects on JMML cell proliferation and colony formation [14, 15].

### Patient-derived induced Pluripotent Stem Cells (iPSCs)

The utilization of iPSCs to construct disease models has become a prevalent approach in the investigation of various disorders [16, 17]. iPSCs possess the remarkable capacity for unlimited self-renewal and differentiation into diverse cell types, thereby offering a replenishable source of pathological research materials for JMML studies. Multiple research groups have successfully generated iPSCs derived from JMML patients and differentiated them into BM cells exhibiting broad phenotypic similarities to primary cells.

In 2013, first-generation iPSCs were established from PB and BM mononuclear cells of patients harboring the *PTPN11-E76K* mutation using STEMCCA lentivirus in Mitchell Weiss laboratory [18]. These iPSCs underwent in vitro differentiation into BM cells displaying leukemia features, including heightened proliferative capacity, sensitivity to GM-CSF, and enhanced phosphorylation of STAT5 and ERK [18]. Subsequently, Tasian et al. employed the same method to generate hematopoietic progenitor cells from iPSCs derived from patients carrying mutations in *CBL-Y371H* and *PTPN11-E76K*, facilitating the first direct comparison of signal abnormalities and drug responsiveness induced by driver mutations [19]. Results revealed that patients with *PTPN11* mutation exhibited significantly higher pERK levels and greater inhibition by MEK inhibitors, while those with *CBL* mutations displayed elevated pJAK2 and pSTAT5 levels and more sensitive to JAK inhibitors [19]. Moreover, iPSCs carrying *PTPN11* mutations have been successfully generated from terminally differentiated skin fibroblasts of patients with Noonan Syndrome (NS) and NS/JMML, subsequently differentiated into hematopoietic lineages [20, 21]. iPSCs derived from NS patients partially exhibited transcriptional and protein expression profiles similar to JMML patients, thereby providing a valuable platform for targeted drug screening [21].

Current research on iPSCs has effectively recapitulated the hallmark characteristics of JMML during hematopoietic differentiation. Given the constraints associated with primary samples, iPSC technology harbors immense potential for elucidating the pathogenesis of JMML and facilitating drug development. Nonetheless, it is imperative to acknowledge the artificial nature of this system and the inherent risk of further transformation during reprogramming. Continued research efforts are warranted to validate the reliability and utility of these models.

## Stable cell line models based on viral transduction

While the establishment of cell lines directly derived from JMML patients remains unsuccessful, cell lines remain indispensable models in the research landscape. To circumvent this limitation, researchers introduce exogenous mutant genes into host cells via retroviral or lentiviral vectors to achieve stable expression of the exogenous gene-encoded protein. However, it is crucial to acknowledge certain limitations associated with this approach in evaluating oncogene function. Firstly, the function of oncogenes might heavily influence by the genetic background of the cell lines which often carry diverse mutation spectra. Secondly, DNA randomly integrates into the target cell genome, resulting in unpredictable position and copy number of exogenous genes. Thirdly, cells usually only introduce one driving mutation, which may not fully represent the complexity of JMML pathogenesis. Despite these limitations, stable cell lines generated through viral transduction remain invaluable tools for dissecting the functional consequences of specific mutations and elucidating underlying molecular mechanisms. They facilitate high-throughput screening of potential therapeutic agents and provide insights into drug resistance mechanisms.

### Ba/F3 cell line

The Ba/F3 cell line is a murine pro-B cell line reliant on interleukin-3 (IL-3) for survival and growth [22]. The introduction of specific oncogenic genes into Ba/F3 cells can confer independent from IL-3, enabling proliferation solely through the activity of the introduced oncogene. In Loh et al. study, the expression of SHP2-D61Y/-E76K mutants enabled Ba/F3 cells to survive independent of IL-3 for a period of 2–3 weeks[23]. However, this survival was not accompanied with significant cell proliferation or abnormal activation of IL-3-induced ERK and AKT signaling pathways [23]. Another study reported that SHP2-E76K expression via retroviral infection enhanced IL-3-induced activation of ERK and AKT, and the proportion of cells entering the S phase in cells [24]. Additionally, expression of SHP2 mutants in Ba/F3 cells through the same method did not reveal any discernible biological differences in another study [25]. We also replicated the above experiments and did not observe IL-3-independent cell survival induced by D61Y and E76K mutants [26]. This is not an unprecedented phenomenon, as JAK2-V617F causes polycythemia vera but also fails to transform Ba/F3 cells [27]. The underlying reasons for this phenomenon remain unclear and warrant further investigation.

Despite the controversy surrounding SHP2 mutations, other mutations identified in JMML, such as *KRAS* [28], *NRAS *[29, 30], and *JAK3 *[31, 32], have been shown to confer IL-3 independent growth on Ba/F3 cells. In the study by Qian et al., they found that approximately one-third of *NRAS* mutations significantly induced IL-3-independent growth and activation of pERK in Ba/F3 cells [30]. This included mutations such as G12D and G12V, which are most commonly found in JMML [30]. It is worth mentioning that Ba/F3 cells have been utilized in the development of personalized treatment approaches for patients. Chao et al. reported a novel CCDC88C-FLT3 fusion gene-driven JMML patient who was resistant to conventional cytotoxic chemotherapy [33]. Here, researchers demonstrated the transformation of this fusion protein using Ba/F3 cells and found the sensitivity to FLT3 inhibitor Sorafenib [33]. Consequently, the patient treated with Sorafenib and achieved cytogenetic remission after 10 weeks and safely undergoing HSCT [33].

### TF-1 cell line

The TF-1 cell line, derived from a patient with erythroleukemia, also represents a valuable model for studying hematopoietic disorders and their associated molecular mechanisms.TF-1 cells rely on GM-CSF or IL-3 for survival and proliferation, making it more suitable for JMML research. In Bunda et al. study, they demonstrated that Cbl-Y371H mutant increased Lyn-dependent AKT pro-survival signaling using TF-1 cells [34]. Jie Wu’s research group showed that expression of the SHP2-E76K mutation in TF-1 cells conferred survival and proliferation without GM-CSF and was mediated by upregulation of ERK1/2 and its downstream effector Bcl-XL [35]. In 2017, they continued to utilize this model to assess the efficacy of the allosteric SHP2 inhibitor SHP099 against common JMML-related *PTPN11* mutations (D61Y, E69K, A72V, and E76K) [36]. While all these mutations were sufficient to transform TF-1 cells, only the E69K exhibited sensitivity to SHP099 [36]. Crystal structure analysis further elucidated the underlying mechanism, revealing minimal interference with the SHP099 binding tunnel caused by the position of the E69K mutation [36]. These experiments particularly highlight the advantages of cell line models in oncogene analysis and drug screening, making them indispensable in targeted drug research.

### HCD-57 cell line

The HCD-57 cell line is a mouse erythroleukemia cell line dependent on erythropoietin (EPO) for survival. Recently, we achieved EPO-independent survival in HCD-57 cells by expressing SHP2-D61Y/E76K using the MSCV-IRES-EGFP retroviral vector [26]. The mutated cells exhibited hallmark features of JMML, including overactivation of the MAPK and PI3K-AKT signaling pathways [26]. Importantly, the mutated cells demonstrated rapidly proliferated in the spleen and BM of immunodeficient mice, providing an excellent model for in vivo testing of drugs targeting aberrant SHP2 signaling[26]. Based on this platform, we screened and identified Sunitinib as selectively inhibiting SHP2-mutant leukemia cells in vitro and in mouse models, offering promising prospects as a new effective treatment strategy for SHP2-mutant JMML in the future [26]. The utility of HCD-57 cells extends to both in vitro drug screening and in vivo preclinical testing, facilitating the identification of potential targeted therapies for this challenging disease.

## Genetically engineered mouse models

The identification of key mutations in JMML has enabled the development of experimental models using genetic engineering methods. An ideal JMML model should express mutation at appropriate times and levels within the hematopoietic lineage to reflect its fetal origin and hematopoietic restriction. Initially, researchers expressed mutations in mouse embryos and found that homozygous mutations were lethal. Subsequently, gene transduction and BM transplantation were used to proliferate mutant cells in recipient mice. However, these models exhibited low disease penetrance and were extremely complex to operate. Currently, the Cre-LoxP system is the primary mouse model for research. This technology effectively addresses issues of embryonic lethality and uncontrolled expression of pathogenic genes. However, it is important to note that many Cre recombinases have activity in multiple tissues. For example, Mx1-Cre is also expressed in endothelial cells, gastrointestinal tract cells, and bone marrow mesenchymal cells. The expression of mutations in non-hematopoietic tissues can exacerbate MPN characteristics and even lead to tumors in other systems impacting the progression of hematologic diseases. Additionally, the high-dose radiation administered to recipient mice can inappropriately exacerbate MPN. Therefore, the reliability of these models needs careful evaluation. The major genetically engineered mouse models mediated by Cre/loxP system and other construction methods are summarized in Tables 1 and 2 respectively.

**Table 1:** Genetically engineered mouse model mediated by Cre/loxP system

Driver	Mouse genetype	Cre inducer	Survival condition	Notes	References
*Nf1*	*Nf1*^*f/f*^, *Mx1-Cre*	pIpC	mOS = 7.5 Months	1. Develops MPD with slow progression, not progressing to acute leukemia 2. Capable of transplantation in lethally irradiated mice	Le et al. [45]
	*Nf1*^*f/f*^, *Mx1-Cre*	pIpC; MOL4070LTR	mOS = 140 Days	1. Virus-induced cooperative mutations cause *Nf1*-deficient mice to progress from MPD to AML 2. AML tumor cells are more dependent on the Ras-MEK signaling pathway	Lauchle et al. [46]
	*Nf1*^*f/f*^, *Tie2-Cre*	NA	Killed at 9 Months	1. Part of the mouse embryos died due to cardiovascular defects 2. The surviving mice developed MPD at 3 months	Gitler et al. [47]
*Kras*	*LSL*-*Kras*^*G12D*^, *Mx1-Cre*	pIpC	mOS = 35 Days	1. Developed penetrant MPD, not progressing to AML 2. Not capable of transplantation in sublethally irradiated mice 3. Mice not induced with pIpCalso developed intermediate severity MPD	Chan et al. [50]
		NA	mOS = 58 Days		
	*LSL*-*Kras*^*G12D*^, *Mx1-Cre*	pIpC	mOS = 84 Days	1. Developed fatal MPD 2. Elevated Ras-GTP levels but no increase in AKT and MEK phosphorylation, and sensitive to GM-CSF	Braun et al. [51]
	*LSL*-*Kras*^*G12D*^, *Flt3-Cre*	NA	mOS = 26 Days	1. Caused fatal MPN, resembling JMML 2. Changes in stem and progenitor cell populations in BM and spleen 3. Activated RAS pathway and sensitive to GM-CSF 4. Capable for transplantation	Tarnawsky et al. [56]
*Ptpn11*	*LSL*-*Ptpn11*^*D61Y*^, *Mx1-Cre*	pIpC	mOS = 45 Weeks	1. Caused fatal MPD; 2. In addition to leukocytosis and hepatosplenomegaly, also appeared anemia 3. Different hematopoietic cell compartments exhibit diverse and distinct effects 4. Failure to transplant *Ptpn11*^*D61Y*^-evoked MPD;	Chan et al. [61]
	*Ptpn11*^*E76K/*+^, *Mx1-Cre*	pIpC	mOS = 28 Weeks	1. After 12–32 week of chronic MPD, *Ptpn11*^*E76K/*+^ mice progressed to the accelerated phase, presenting with AML, B-ALL and T-ALL 2. *Ptpn11*^*E76K*^ mutation has non–lineage-specific effects on malignant transformation of hematopoietic cells and initiates acute leukemias at various stages of hematopoiesis	Xu et al. [63]
	*Ptpn11*^*E76K/*+^, (1)* LysM-Cre*; (2) *LCK-Cre*; (3) *CD19-Cre*;	NA	mOS = 170 Days	1. LysM-Cre, LCK-Cre, and CD19-Cre transgenic mice used to generated lineage-specific knock-in mice in GMPs, CD4^−^/CD8^−^ stage T cells and in pro–/pre–B stage B cells, respectively 2. *Ptpn11*^*E76K/*+^*/LysM-*Cre^+^ (40%), *Ptpn11*^*E76K/*+^*/LCK-*Cre^+^ (53%), and *Ptpn11*^*E76K/*+^*/CD19-*Cre^+^ (44%) mice subsequently developed AML, T-ALL, and B-ALL, respectively 2. These mice also transplantable in primary and secondary recipient mice	Xu et al. [63]
	*Ptpn11*^*E76K/*+^, (1) *Nestin-Cre*; (2) *VE-Cadherin* -*Cre-ER*^*T2*^; (3) *Prx1-Cre*; (4) *Lepr-Cre*; (5) *Osx-Cre*; (6) *Oc-Cre*;	NA	Age of mice euthanized: (1) 7–14 Months (2) 11–18 Months (3) 5–10 Months (4) 13–17 Months (5) 5–8 Months (6) 11–18 Months	1. Developed MPN: *Ptpn11* mutation expression in MSPCs (1), mesenchymal cells (3), leptin receptor^+^ mesenchymal cells (4), and osteoprogenitors (5) 2. MPN not in differentiated endothelial cells (2) or osteoblasts(6) 3. *Ptpn11* mutations in the BM microenvironment have pathogenic effects on resident HSCs, promoting/inducing leukaemogenesis	Dong et al. [64]
	*Ptpn11*^*E76K/*+^*,* (1) *Flt3-Cre*; (2) *Csf1r-MER*^+^-*Cre*	(1) NA (2) Tamoxifen	(1) mOS = 66 Weeks (2)mOS = 67 Weeks for 4wk Tam mOS = 62 Weeks for E14.5 Tam	1. Caused indolent MPN 2. Cre activity is specifically expressed in the hematopoietic system, which will not exacerbated by non-hematopoietic oncogene expression 3. Simulates the fetal origin of JMML	Tarnawsky et al. [62]
*Nras*	*LSL*-*Nras*^*G12D*^, *Mx1-Cre*	pIpC	NA	1. Mice do not have MPD disease phenotypes 2. After the second transplantation, the recipient mice showed CMML phenotype 6 months later	Wang et al. [66]
	*LSL*-*Nras*^*G12D*^, *Mx1-Cre*	pIpC	mOS = 363 days	1. Died of differ hematologic disorders with myeloproliferation, including MPD 2. In C57BL6/129Sv/jae strain background the mOS is 363 days and in C57Bl/6 mice the mOS is 588 days	Li et al. [67)
		pIpC, MOL4070LTR	mOS = 200 days	1. Caused AML 2. *Nras*^*G12D*^AMLs can be inhibit by MEK inhibitor PD0325901 compared with normal myeloid progenitor growth	
	*LSL*-*Nras*^*G12D*^, *Mx1-Cre*	NA	Age of mice euthanized: 4–8.5 months	1.Develop MPD faster and severer than those who injected with pIpC 2.Capable for secondary transplantation	Wang et al. [68]
*Cbl*	Cbl^f/f^, *VAV1-Cre*;	NA	mOS = 12 days	1. Develop a very severe MPD at 10 Days 2. Capable for transplantation in lethally irradiated recipients 3. Leukemic stem cells were most highly enriched in neonatal liver, while BM, spleen, and thymus were hypo-cellular	An et al. [72]
	Cbl^f/f^, *Cre*^*ERT2*^	Tamoxifen	mOS = 60 days (Recipient mice)	1. Recipient mice developed aggressive CMML-like disease 2. JAKi can inhibit the development of *Cbl*^*−/−*^	Lv et al. [73]

*mOS* Median overall Survival, *MPD* Myeloproliferative disorder, *AML* Acute myelogenous leukemia, *MPN* Myeloproliferative neoplasm, *JMML* Juvenile myelomonocytic leukemia, *BM* Bone marrow, *GM-CSF* Granulocyte–macrophage colony-stimulating factor, *B-ALL* B cell acute lymphoblastic leukemia, *T-ALL* T cell acute lymphoblastic leukemia, *GMPs* Granulocyte–Macrophage Progenitors, *CMML* Chronic myelomonocytic leukemia

**Table 2:** Genetically engineered mouse model mediated by other construction method

Driver	Mouse genetype	Construction method	Survival condition	Notes	References
*Nf1*	*Nf1*^*−/−*^, *Nf1*^*+/−*^	Homologous recombination in ES; Adoptive transfer of fetal liver cells	Homozygous embryos are lethal	1. *Nf1*^*−/−*^ mouse embryos die at E12-14 2. 10% *Nf1*^*+/−*^ mice develop MPD during the second year of life 3. Transplantation of *Nf1*^*−/−*^ fetal liver hematopoietic cells into irradiated mice induces MPD (18–30 weeks post-transplant)	Brannan et al. [43]; Largaespada et al. [13]; Zhang et al. [42, 44]
*Kras*	*Kras*^*V14I/V14I*^; *Kras*^*V14I/*+^	Homologous recombination in ES	*Kras*^*V14I/V14I*^: mOS = 36 Weeks; *Kras*^*V14I/+*^: mOS = 62 Weeks	1. *Kras*^*V14I/V14I*^ animals had significant perinatal lethality 2. *Kras*^*V14I/V14I*^ and *Kras*^*V14I/*+^ displayed MPD 3. These mice recapitulates NS	Hernandez-Porras et al. [49]
*Ptpn11*	*Ptpn11*^*D61G/ D61G*^*;* *Ptpn11*^*D61G/*+^	Homologous recombination in ES	*Ptpn11*^*D61G/ D61G*^ embryo lethal; *Ptpn11*^*D61G/*+^ embryo lethal 50%	1. *Ptpn11*^*D61G/D61G*^ embryos die in mid-gestation 2. Survival *Ptpn11*^*D61G/*+^mice developed a mild MPD at 5 months but do not affect survival in 10 months 3. These mice recapitulates NS	Araki et al. [60]
	*Ptpn11*^*D61Y*^*; Ptpn11*^*E76K*^	BMT	mOS≈6–7 months,	1. Mice had severe MPD (∼60%), T-ALL/lymphoma (∼20%) 2. T-ALL/lymphoma mice were transplantable, while MPD mice failed	Mohi et al. [25]
*Nras*	*Nras*^*G12D*^	BMT	mOS = 35–55 days	1. Mice rapidly developed CMML or AML 2. Capable for secondary transplantation	Parikh et al. [65]
*Cbl*	*c-Cbl *^*A/A*^*,* *c-Cbl *^*A/−*^ (A:C379A)	Homologous recombination in ES	*c-Cbl *^*A/A*^ embryo lethal; *c-Cbl *^*A/−*^: mOS = 47.5 weeks	1. *c-Cbl *^*A/−*^ mice exhibit MPD and progressing thymus atrophy 2. Capable for secondary transplantation	Thien et al. [70] Rathinam et al. [71]

*ES*: Embryonic Stem Cell; *MPD* Myeloproliferative disorder, *mOS* Median overall Survival, *BMT* gene transduction/bone marrow transplantation, *AML* Acute myelogenous leukemia, *T-ALL* T cell acute lymphoblastic leukemia, *CMML* Chronic myelomonocytic leukemia

### Nf1 mutation-driven mouse model

The inactivation on the *NF1* allele is a common autosomal dominant genetic disorder, increasing the risk of malignant bone marrow diseases in children (but not adults) by 200–500 times, particularly for JMML [37, 38]. Studies on primary samples from JMML patients have confirmed that *NF1* acts as a tumor suppressor by negatively regulating Ras proteins in immature hematopoietic cells [37, 39, 40]. In Nf1 knockout mouse models, *Nf1*^*−/−*^ embryos die mid-gestation (E12–E14) due to cardiac defects, while approximately 10% of heterozygous *Nf1*^*+/−*^ mice spontaneously develop JMML-like MPD and exhibit loss of the wild-type allele during the second year of life [41–43]. Furthermore, researchers have adoptive transferred fetal liver hematopoietic cells from *Nf1*^*−/−*^ mice into irradiated recipient mice, inducing Ras hyperactive JMML-like MPD in the recipients [13, 42]. These models provided the first mouse tools for studying the mechanisms by which *Nf1* leads to JMML. However, this fetal liver cell transplantation model has important limitations [44]. Large number of cells are required to induce the disease phenotype. And in the later stages, the mutant cells form stable chimeras with wild-type cells without excessive expansion. Moreover, mutant cells significantly expand in the spleen of recipient mice but behave normally in the BM [44].

In subsequent research, researchers have utilized the Cre-LoxP system to construct conditional gene knockout mouse models. In Le et al. study, they found that this method could induce mice to develop MPD, with phenotypes including leukocytosis, splenomegaly, and hypersensitivity to GM-CSF [45]. This model exhibited a relatively slow disease progression, and their blood cell counts did not fluctuate dramatically [45]. Although the induced mice developed MPD and supported secondary transplantation, they did not progress to acute leukemia [45]. In further research, Lauchle et al. used simultaneous injection of MOL4070LTR (a retrovirus that induces murine leukemia) and pIpC to knockout *Nf1* and induce cooperative mutations, causing mice to progress from MPD to AML [46]. Additionally, researchers employed *Tie2-Cre* mice to construct *Nf1* gene knockout models. However, since Cre recombinase is expressed in all endothelial cells and hematopoietic lineages, some mice died during embryonic development due to cardiac defects [47]. The surviving mice exhibited MPD at 3 months, displaying defects very similar to JMML [47].

### Kras mutation-driven mouse model

*KRAS* is a highly prevalent oncogene found in pancreatic, colorectal, and lung cancer patients. Johnson et al. introduced the *KRas*^*G12D*^ mutation into embryonic stem (ES) cells, resulting in mice that exhibited multifocal tumors at various stages of progression, with lung tumors being the most common [48]. Additionally, when the *KRas*^*V14I*^ mutation was expressed in ES cells, homozygous mice showed significant perinatal mortality, and the survivors displayed NS characteristics and MPD [49]. In subsequent studies, researchers predominantly used the Mx1-Cre/LoxP system to restrict the expression of mutations to hematopoietic cells. Braun et al. and Chan et al. induced *Kras*^*G12D*^ expression in mice at 21 days and 4–7 weeks of age, respectively [50, 51]. Both groups of mice exhibited 100% penetrance of MPD, with mortality occurring between 4 and 16 weeks, and showed monocytosis, BM infiltration, and sensitivity to growth factors [50, 51]. Moreover, they demonstrated that spontaneous Cre activity in mice, even without pIpC induction, was sufficient to trigger MPD with similar symptoms, albeit milder overall [50]. It is important to note that while Cre is primarily expressed in the hematopoietic system, it is also expressed in the liver, spleen, lung endothelium, and kidneys [52]. Consequently, some mice developed lung adenomas, esophageal squamous papillomas, and other tumors [50]. These additional pathologies should be considered when evaluating the MPN phenotype in this model. In subsequent research, Staffas et al. proposed that the rapid mortality observed in primary mice was not due to MPD, but rather due to intestinal bleeding and severe anemia caused by the expression of the mutation in non-hematopoietic cells. They also demonstrated that this model, when transplanted into wild-type mice, could develop into T-ALL [53]. Other studies used *LysM-Cre* and *Vav-Cre* to induce *Kras*^*G12D*^ expression in utero, resulting in lung adenocarcinoma and prenatal lethality [54, 55].

Tarnawsky et al. addressed these challenges by generating *Flt3Cre/LSL*-*Kras*^*G12D/*+^ mice achieving precise temporal and spatial expression of Kras^G12D^ without interference from lung cancer or T-ALL [56]. Firstly, Flt3Cre restrictively expresses in the hematopoietic system [57]. Secondly, Flt3Cre activity initiates at embryonic day 10.5, effectively modeling the fetal origin of JMML [56]. *Flt3Cre*/*Kras*^*G12D*^ mice exhibit progressive MPN with anemia, thrombocytopenia, and hepatosplenomegaly starting from 2 weeks after birth. MPN progresses rapidly with a median survival of only 26 days [56]. Bone marrow progenitor cells from these mice show hypersensitivity to GM-CSF and continue to induce JMML-like MPN in recipient mice post-transplantation [56].

### Ptpn11 mutation-driven mouse model

*PTPN11* mutations are most commonly found in JMML and NS, and are also present in B-ALL and AML, as well as solid tumors such as lung adenocarcinoma and colorectal cancer [25, 58]. Transducing mutant *Ptpn11* (E76K and D61Y) into mouse BM leads to hypersensitivity of hematopoietic cells to GM-CSF [59]. These mutations transduced into mouse BM and transplanted into lethally irradiated recipient mice, 60% of the mice develop severe MPD, and the remaining mice exhibit T-ALL/lymphoma [25]. Targeting the *Ptpn11*^*D61G*^ mutation into ES cells and injecting them into mouse blastocysts results in embryonic lethality due to cardiac defects in homozygous D61G mutants [60]. The number of heterozygous mice born is only 50% of the expected number. The surviving mice are small in stature and develop mild MPD at 5 weeks of age, which does not lead to death [60]. These models provide initial insights into the pathogenesis of PTPN11-induced MPD.

Chan et al. generated the *Ptpn11*^*D61Y/*+^ /*Mx1-Cre*^+^ mouse model [61]. These mice develop fatal MPD, characterized by granulocytosis, monocytosis, hepatosplenomegaly, extramedullary hematopoiesis, and severe anemia, and without the presence of other tumors [61]. The mutation affected various hematopoietic cell populations, including HSCs, myeloid progenitors, and erythroid progenitors [61]. Tarnawsky et al. used *Csf1r-MCM-Cre* and *Flt3Cre* to create *Ptpn11*^*E76K*^-mediated hematopoietic-specific expression models [62]. Flt3Cre is active in utero at E10.5, and Csf1r-MCM is induced by tamoxifen [62]. These models showed slower MPN progression, with fetal expression leading to relatively faster disease progression [62].

Qu et al.’s research team made significant contributions to understanding Ptpn11’s leukemogenic role. In 2011, they restricted the *Ptpn11*^E76K/+/^*Mx1-Cre*^+^ mouse model and found that the mutation quickly led to MPD with abnormal activation of hematopoietic stem and myeloid progenitor cells [63]. Importantly, these mice subsequently develop various acute leukemias, including AML, T-ALL, and B-ALL [63]. They also generated lineage-specific knock-in mice using *LysM-Cre*, *LCK-Cre*, and *CD19-Cre* mice, with E76K mutation restricted to GMPs, T cells, and B cells, respectively [63]. *Ptpn11*^E76K/+^/*LysM-Cre*^+^ mice show MPD at weaning, which then progresses to AML, and other mice develop T-ALL and B-ALL after four months, demonstrating the mutation's non-lineage/phase-specific impact on LSC development [63]. In 2016, they further restricted Ptpn11^E76K^ expression to BM mesenchymal stem/progenitor cells (*Nestin-cre*^+^), endothelial cells (*VE-Cadherin*-*Cre*-*ER*^*T2*^), mesenchymal cells (*Prx1-Cre*^+^), leptin receptor^+^ mesenchymal cells (*Lepr-Cre*^+^), osteoprogenitors (*Osx1-Cre*^+^), and osteoblasts (*Oc-Cre*^+^) to explore the role of mutation in microenvironment [64]. The results indicated that mutations in BM mesenchymal stem/progenitor cells and osteoprogenitors induced MPN [64]. Mechanistic studies revealed that Ptpn11^E76K^ in the microenvironment led to excessive production of the chemokine CCL3, recruiting monocytes to the HSC niche, resulting in overactivation of adjacent HSCs and inducing/promoting MPN in mice [64].

### Nras mutation-driven mouse model

*NRAS* gene mutations frequently lead to melanoma, colorectal cancer, MDS, and myeloid tumors. Knocking out *Kras* in mice results in embryonic lethality, while *Nras*-deficient mice are viable [65]. Expressed *Nras*^*G12D*^ in mouse BM cells and transplanted into lethally irradiated recipients, the mice rapidly develop fatal CMML/AML-like hematologic diseases and support secondary transplantation experiments [65]. This demonstrates the oncogenic potential of Nras mutations.

In 2010, Wang et al. established the *Nras*^*G12D/*+^*/Mx1-Cre*^+^ mouse model [66]. However, the expression of Nras-G12D did not result in any disease phenotype [66]. They subsequently performed secondary transplantation, the recipient mice died from CMML-like disease after six months [66]. In a separate study, Li et al. also used the *Nras*^*G12D/*+^*/Mx1-Cre*^+^ model [67]. These mice exhibited indolent MPD and ultimately succumbed to various hematologic tumors [67]. So, Wang et al. continued to investigate the reasons behind these differences, and found the mice developed fatal MPD without pIpC induction [68]. Therefore, it may be that Cre enzyme activity expressed in other organs interferes with MPD development in mice, which is similar to the phenotype that occurs in *Kras* model mice.

### Cbl mutation-driven mouse model

CBL proteins are members of the ubiquitin ligase (E3) family and negatively regulate receptor tyrosine kinase signaling pathways. There are three main types of Cbl in mammalian: Cbl (also known as c-Cbl), Cbl-b, and Cbl-c[69]. Mutations in Cbl-b and Cbl-c are rare in myeloid tumors, while mutations in c-Cbl lead to various of myeloid tumors, mainly including JMML and CMML [69]. Disease-causing mutations in c-Cbl often occur in the RING finger domains, which is critical for regulating the function of the E3 function of Cbl. In gene knock-in mice, homozygous mutations at the C379A site in *c-Cbl* result in embryonic lethality [70]. Therefore, single-copy mutant *c-Cbl*^*A/−*^ mice is commonly used to study *Cbl* functions. Rathinam et al. demonstrated that *c-Cbl* knockout mice *c-Cbl*^*−/−*^ develop very mild MPD, while *c-Cbl *^*A/−*^ mice develop aggressive myeloid leukemia [71].

An et al. created a hematopoietic-specific Cbl-deficient mouse model by inducing *Cbl/Cbl-b* double knockout using *VAV1-Cre* [72]. This mouse model develops JMML-like MPD around 10 days after birth, with rapid progression closely mirroring the natural course of JMML [72]. The mice are capable of secondary transplantation, although the disease progresses more slowly in the recipients [72]. Utilizing a method involving the isolation of HSPC cells from *Cbl*^*f/f*^, *Cre*^*ERT2*^ mice followed by transplantation into recipient mice and subsequent injection of 4-hydroxytamoxifen to induce Cre expression, a relatively quiescent hematopoietic system-specific Cbl-deficient mouse model can be generated. This model has a median survival of 60 days and exhibits aggressive CML with symptoms that are similar to JMML, providing a more operational model for drug and pathogenesis studies [73].

## Patient-derived xenografts (PDX) models

Genetically engineered mice indeed induce MPD phenotypes, but they remain murine leukemias. Key disease features of JMML, such as recurrent monosomy 7 or elevated fetal hemoglobin, cannot be recapitulated in these models [74]. Moreover, transgenic animal models are often driven by a single etiology, making it difficult to mimic the complexity of human tumor initiation. PDX models involve directly implanting tumor cells from patients into immunodeficient mice. These approach retains tumor heterogeneity and more closely resemble the tumor microenvironment within patients. Several studies have reported high fidelity of PDX models in genomics, transcriptomics, and histology [75]. And related researches have demonstrated that the predictability of clinical treatment response in PDX exceeds 80%, compared to only about 5% in traditional models [76, 77]. The success rate of PDX model establishment is associated with tumor type, recipient mouse strain, and transplantation method. For example, colorectal cancer transplants have high survival rates in nude mice, while establishing PDX models of hematologic malignancies requires severely immunodeficient mice [77]. The main JMML-PDX models are listed in Table 3.

**Table 3:** JMML-PDX models

Murine strain	Primary cells		Transplantation protocols			End-point **(**week)	Notes	References
	Source	Type	Age (week)	Radiation dose	Injection mode and cell number			
SCID	PB, BM, Spleen	Percoll- enriched leukemic cells	7–8	350–400 cGy	IV, 0.1– 4 × 10^7^	2–4	1.Recapitulated the patient disease phenotype 2.Capable of secondary transplants 3.GM-CSF were necessary	Lapidot et al. [78]
NOD/SCID	BM, Spleen	MNC	4–6	200 cGy on day 1 and 3	IV, 2– 3 × 10^7^, on day1 and 3	6	1. JMML patient cells transplanted into mice showed engraftment rates of less than 20% at 2 weeks, over 80% at 4 weeks, and a slight increase at 6 weeks	Iversen et al. [79]
NOD/SCID/ γc^−/−^	BM, Spleen	MNC	5	–	IV, 0.01–1 × 10^7^	12	1. A minimum dose of 0.3 × 10^^^7 primary cells were needed to detect human CD45^+^ cells in mouse bone marrow after 12 weeks 2. Patient cells proliferate in mice and differentiate into granulocytes, monocytes, erythrocytes, B cells, T cells, and NK cells	Nakamura et al. [81]
Rag2^−/−^γc^−/−^ BALB/c	Spleen	MNC, and MNC further depleted CD3 + T cells	Newborn	2.5 Gy	Intrahepatically injection, 1 × 10^6^	> 20	1. 4 patient samples were transplantable; 1 failed. Different engraftment levels in mice, no difference between techniques 2. T cells may cause graft-versus-host disease, leading to transplant failure, depending on sample differences 3. Mice recapitulated key JMML features 4. Tumor cells in model mice supported serial transplantation, lasting 1.5 years in vivo	Krombholz et al. [82]
			5	3 Gy	IV, 5 × 10^6^			
NSGS	BM, PB	MNC	6–10	200–250 cGy,	IV, 1.3–4.1 × 10^6^	mOS = 43d	1. The PDX model accurately reflected the patients' clinical features, with bone marrow monocyte proliferation causing splenomegaly and alveolar hemorrhage in 3 of 4 patients 2. Recipient mouse phenotypes correlated with patient clinical courses, with the most aggressive samples leading to the fastest PDX model establishment and shortest survival times	Yoshimi et al. [83]
NSG	BM	CD34^+^ cells, and HSPC subfractions	8–12	350–375 cGy	Tibia injection, 15 × 10^3^	12	1. Both NSG and NSGS mouse models replicate the clinical features of JMML pathology 2. NSGS mice show higher engraftment rates (NSGS: 9/10, NSG: 8/12), faster disease progression, and more pronounced phenotypes 3. Both models retain the primary samples' mutation types, frequencies, and clonal structures, with secondary transplants showing similar clonal compositions 4. HSC/MPP and more mature progenitors like LMPP/CMP/GMP can proliferate in the mouse bone marrow	Caye et al. [84]
NSGS						6		

*BM* Bone marrow, *PB* Peripheral blood, *IV* Tail vein injection, *GM-CSF* Granulocyte–macrophage colony-stimulating factor, *MNC* Mononuclear cell (cells were separated by centrifugation on Ficoll Hypaque), *HSC* Hematopoietic stem cells, *MPP* Multipotential progenitors, *LMPP* Lymphoid Myeloid progenitors, *CMP* Common myeloid progenitors, *GMP* Granulocyte–Macrophage Progenitor

The earliest establishment of JMML-PDX occurred in 1996. Lapidot et al. transplanted leukemia cells collected from JCML (now known as JMML) patients' BM, PB, and spleen into irradiated SCID mice and stimulated with GM-CSF [78]. The patient leukemia cells proliferated extensively in the mouse BM, exhibiting hepatosplenomegaly, anemia, and thrombocytopenia [78]. High levels of GM-CSF were necessary here, increasing the implantation levels from 33.3% to 100% [78]. Iversen et al. transplanted JMML patient BM mononuclear cells into irradiated SCID/NOD mice, and the leukemia cells constituted a major portion of the mouse BM after 4 weeks [79, 80]. In 2005, Nakamura et al. developed a novel JMML xenograft model using NOD/SCID/γc^−/−^ mice, which lack T, B, and NK cells [81]. This model demonstrated that JMML originates from multi-lineage HSC, as human granulocytes, monocytes, erythrocytes, B cells, T cells, and NK cells were detected in the mice [81].

The aforementioned xenograft models still face challenges with leukemia cell engraftment, requiring GM-CSF stimulation or large numbers of primary cells inputs [74]. Additionally, the early PDX mouse models quickly developed severe disease phenotypes, limiting the detailed analysis of natural disease progression and comprehensive drug evaluation experiments. Recently, more immunodeficient mouse models have been developed and used in JMML research, providing more ideal PDX models. Particularly, the extremely immunodeficient NOD/SCID-IL2Rγ^null^ (NSG) and their second-generation strain NSGS (expressing human GM-CSF, IL-3, and SCF) have become the preferred models for constructing JMML-PDX.

Krombholz et al. used Rag2^−/−^γc^−/−^ BALB/c mice as recipient mice to create a JMML xenograft model displaying a chronic disease course, with a median survival of over 20 weeks [82]. Importantly, tumor cells from these model mice supported serial transplantation, with JMML cells maintained in vivo for 1.5 years [82]. Yoshimi et al. achieved efficient engraftment by transplanting JMML patient mononuclear cells into irradiated NSGS mice with relatively low cell numbers [83]. This model better recapitulated the clinical characteristics of JMML patients, including splenomegaly and alveolar hemorrhage caused by excessive proliferation of bone marrow monocytes infiltrating organs [83]. Furthermore, the phenotype of recipient mice appeared to correlate with the clinical course of the patients, as the most aggressive patient samples maintained their clonal structure better in xenografts, leading to earlier death in mice and an aggressive phenotype in secondary recipient mice [83]. Caye et al. conducted a comprehensive evaluation of JMML disease recapitulation, clonal composition, and clonal evolution in immunodeficient mice by transplanting JMML patient BM-CD34 + cells into NSG and NSGS mouse models [84]. Their results demonstrated that both mouse models could recapitulate clinical features of JMML, such as splenomegaly, with engraftment dynamics and levels related to the initial JMML mutations. However, NSGS mice exhibited higher engraftment rates, faster disease progression, and more pronounced disease phenotypes, highlighting the hypersensitivity of JMML to GM-CSF [84]. Additionally, both models preserved the mutational characteristics of the primary samples, including mutation types, mutation frequencies, and clonal structures, with secondary transplanted mice displaying similar clonal compositions. Furthermore, not only HSC/MPP but also more mature progenitors could proliferate in the mouse bone marrow [84].

## Zebrafish models

Although the number of studies utilizing zebrafish models for JMML research is relatively limited at present, they hold significant promise for extensive exploration. Zebrafish share a high degree of similarity with humans in hematopoietic genetic and molecular mechanisms, with hematopoietic signaling pathways being highly conserved [85]. Many mutations associated with human blood diseases have been successfully replicated in zebrafish, resulting in phenotypes resembling human diseases [86, 87]. Zebrafish are robust breeders and develop rapidly, making them particularly suitable for large-scale drug screening [86]. Moreover, they are amenable to various genetic manipulation techniques [88]. Notably, zebrafish are ideal for studying embryonic and larval hematopoiesis due to their small size and transparency during early developmental stages, allowing for simple high-resolution optical imaging of live animals to monitor the initial stages of disease onset [86].

Some studies have explored the effects of mutations on zebrafish development by microinjecting mRNA into single-cell stage embryos. Researchers found that the expression of *ptpn11*^*D61G*^ mutant in zebrafish embryos disrupted cell movements during gastrulation and resulted in craniofacial and cardiac defects [89, 90]. Additionally, zebrafish models offer the advantage of rapid translation from gene function studies to therapies, particularly using plasmid injection into embryos, with analysis possible 48 h post-injection. A novel tandem repeat sequence mutation occurring in *NRAS* was identified in a patient with unclassified MDS/MPN was tested in zebrafish, resulting in marrow proliferation phenotypes reversed by MEK inhibition [91]. Microinjection methods, however, cannot transmit mutations to the next generation, lack tissue and developmental stage specificity, and exhibit transient expression (within the first 5 days of development) [92]. Therefore, more stably methods have been employed. Solman et al. successfully introduced the *shp2*^*D61G*^ mutation into zebrafish using CRISPR/Cas9 technology [93]. Zebrafish stably expressing the D61G mutant protein exhibited typical NS features and MPN-like hematopoietic defects associated with JMML, including myeloid expansion, mild anemia, and thrombocytopenia [93]. The transcriptomes of patients and zebrafish HSPCs showed similar expression patterns, particularly upregulation of genes associated with inflammation [93]. Additionally, Le et al. developed a heat shock-inducible Cre/Lox system for conditional stable expression of *kRAS*^*G12D*^ in zebrafish, leading to MPD characterized by expansion of granulocytic and monocytic/macrophage lineages [94].

## Conclusions

Due to the high mortality, it is urgent to develop practical experimental models that more closely reflect JMML biology. However, most in vitro studies are often limited to 2D systems and animal models will always differ from the human context. Researchers have reported the generation and characterization of a novel patient-derived three-dimensional in vitro JMML model, sustaining the long-term proliferation of JMML cells with stem cell features and patient-specific hallmarks [95]. These structures are able to contain key hematopoietic niche elements and support active endogenous hematopoiesis, as well as the growth and survival of hematopoietic cells from adult donors, including malignant cell types that are difficult to grow and study ex vivo. This system can provide scalable for mechanism research and drug development and highly operational model of the human body, and it must be the development direction of the future.

## Data Availability

No datasets were generated or analyzed during the current study.

## Abbreviations

HSCs: Hematopoietic stem and progenitor cells
MPN: Myeloproliferative neoplasm
PB: Peripheral blood
BM: Bone marrow
HSCT: Hematopoietic stem cell transplantation
GM-CSF: Granulocyte–macrophage colony-stimulating factor
iPSCs: Patient-derived induced pluripotent stem cells
NS: Noonan Syndrome
IL-3: Interleukin-3
EPO: Erythropoietin
MPD: Myeloproliferative disorder
ES: Embryonic Stem Cell
T-ALL: T cell acute lymphoblastic leukemia
B-ALL: B cell acute lymphoblastic leukemia
CML: Chronic myelogenous leukemia
AML: Acute myelogenous leukemia
